# Hepatitis C Virus Induced Endothelial Inflammatory Response Depends on the Functional Expression of TNFα Receptor Subtype 2

**DOI:** 10.1371/journal.pone.0113351

**Published:** 2014-11-24

**Authors:** Joachim Pircher, Thomas Czermak, Monika Merkle, Hanna Mannell, Florian Krötz, Andrea Ribeiro, Volker Vielhauer, Jonathan Nadjiri, Erik Gaitzsch, Markus Niemeyer, Stefan Porubsky, Hermann-Josef Gröne, Markus Wörnle

**Affiliations:** 1 Medizinische Klinik und Poliklinik I, Klinikum der Universität München, München, Germany; 2 Walter Brendel Centre of Experimental Medicine and Munich Heart Alliance, Ludwig Maximilians University München, München, Germany; 3 Medizinische Klinik und Poliklinik IV, Innenstadt, Klinikum der Universität München, München, Germany; 4 Department of Gynecology, Klinikum rechts der Isar, Technische Universität München, München, Germany; 5 German Cancer Research Center, Heidelberg, Germany; SRI International, United States of America

## Abstract

In hepatitis C virus (HCV) infection, morbidity and mortality often result from extrahepatic disease manifestations. We provide evidence for a role of receptors of the innate immune system in virally induced inflammation of the endothelium in vitro and in vivo. Corresponding to the in vitro finding of an HCV-dependent induction of proinflammatory mediators in endothelial cells, mice treated with poly (I:C) exhibit a significant reduction in leukocyte rolling velocity, an increase in leukocyte adhesion to the vessel wall and an increased extravasation of leukocytes. HCV directly promotes activation, adhesion and infiltration of inflammatory cells into the vessel wall by activation of endothelial viral receptors. Poly (I:C) induces the expression of TLR3 in vivo and hereby allows for amplification of all of the aforementioned responses upon viral infection. Proinflammatory effects of viral RNA are specifically mediated by TLR3 and significantly enhanced by tumor necrosis factor alpha (TNFα). HCV-RNA induces the endothelial expression of TNFα and TNFα receptor subtype 2 and we provide evidence that leucocyte adhesion and transmigration in response to activation of viral RNA receptors seem to depend on expression of functional TNFR2. Our results demonstrate that endothelial cells actively participate in immune mediated vascular inflammation caused by viral infections.

## Introduction

More than 170 million people worldwide are chronically infected with the hepatitis C virus (HCV), which is responsible for over 1 million deaths resulting from cirrhosis and primary liver cancers [1]. Besides liver disease, HCV infection is frequently associated with a variety of autoimmune phenomenona with extrahepatic manifestations including cryoglobulinemia [2], [3], renal disease [4] and vasculitis [2], [3], [5], [6], [7]. Extrahepatic manifestations are often severe and contribute significantly to morbidity and mortality. We have previously shown the impact of activation of viral receptors of the innate immune system, particularly TLR3, on HCV-associated glomerulonephritis [8], [9], [10]. Here we provide evidence for a role of receptors of the innate immune system in virally induced inflammation of the endothelium.

Toll-like receptors (TLR) are an essential part of the innate immune system. TLR recognize conserved pathogen-associated molecular patterns (PAMP) and are expressed on immune cells but also on a number of non-immune cells. TLR recognize molecular patterns associated with microbial pathogens and induce an immune response [11]. TLR3 specifically binds dsRNA of viral origin as well as polyriboinosinic:polyribocytidylic acid (poly (I:C)), a synthetic analogue of viral dsRNA [12]. Besides TLR3, the helicase retinoic acid-inducible gene I (RIG-I) and melanoma differentiation-associated gene 5 (MDA5) may also act as sensors of viral infections by recognition of viral dsRNA [13], [14], [15]. Here we show that both poly (I:C) and HCV-RNA isolated from patients infected with the Hepatitis C virus induce the endothelial expression of proinflammatory cytokines, chemokines and type I interferons as well as adhesion molecules. These effects of viral RNA on endothelial cells are specifically mediated by TLR3. Furthermore, we demonstrate an immunomodulatory role of the TNFα/TNF receptor system in HCV-associated vasculitis with a potential impact on the development of new therapeutic strategies.

## Materials and Methods

Human microvascular endothelial cells (HMEC) were provided by Ades et al. [16] and cultured in M199 medium supplemented with 10% FCS, 10% endothelial growth medium (PromoCell, Germany) and 1% penicillin/streptomycin. Human umbilical vein endothelial cells (HUVEC) were isolated and cultured as described previously [17]. The procedure was approved by the university ethics review board (Ethikkommission der Medizinischen Fakultät der Ludwig-Maximilians-Universität). Written informed consent for the collection and generation of the cell lines was obtained. Cytokines were used in a concentration of 25 ng/ml (TNFα), 10 ng/ml (IL-1β) and 20 ng/ml (IFN-γ). For cytokine prestimulation experiments, HMEC were incubated with a combination of TNFα, IL-1β and IFN-γ for 12 hours, washed with PBS, left in culture medium for 6 hours and washed again with PBS. Subsequently, HMEC were incubated with growth medium (control) or medium containing poly (I:C). For poly (I:C) prestimulation experiments, HMEC were incubated with poly (I:C) (10 µg/ml) for 24 hours, then washed with PBS, and subsequently incubated with TNFα for different time intervals as indicated. For each stimulation experiment controls were performed in parallel using culture medium alone.

### Animals

Animal experiments were performed in wild-type (WT) and TNFα receptor subtype 2-deficient (TNFR2^-/-^) C57BL/6 mice. WT mice were purchased from Charles River (Germany) and TNFR2^−/−^ mice were originally obtained from the Jackson Laboratory (USA). Surgical procedures were performed under short-term anesthesia induced by a single intraperitoneal injection of midazolam 3 mg/kg (Ratiopharm, Germany), fentanyl 0.03 mg/kg (CuraMED Pharma, Germany), and medetomidinhydrochloride 0.3 mg/kg (Pfizer, Germany; produced by Orion Pharma, Finland) diluted in 0.9% NaCl. After the experiments, the animals were killed by injection of an overdose (2 g/kg) of sodium pentobarbital (Merial, Germany). All experiments were conducted in accordance with the German animal protection law and approved by the district government of Upper Bavaria (approval reference number AZ 55.2-1-54-2531-162-08) and the Institutional Animal Care and Use Committee (IACUC). The investigation conforms to Directive 2010/63/EU of the European Parliament. Due to the narrow species restriction of HCV which allows infection of humans and chimpanzees only and the resulting lack of non-chimeric small animal models which would allow for direct studies of hepatitis C virus (HCV), stimulation experiments were performed with intraperitoneal injection of poly (I:C) as indicated.

### Mouse blood cell counts

Mouse blood was collected via a carotid catheter and EDTA used as anticoagulant. Blood cell counts were measured using a Beckmann Coulter Counter (Beckman Coulter, Germany).

### Spleen weight

Immediately after scarification of the animals the spleen was isolated and separated from the surrounding connective tissue. Weight of the organ was measured using a special accuracy weighing machine from Acculab (Sartorius AG, Germany) and normalized to the body weight of the respective animal.

### Intravital microscopy in the mouse cremaster muscle

Leukocyte-endothelium-interaction in vivo was assessed in postcapillary venules of the mouse cremaster muscle. Mice were anesthetized and the right carotid artery was cannulated for administration of fluorescent microspheres to determine blood flow velocity. The cremaster muscle was prepared for intravital microscopy as originally described by Baez et al [18]. Throughout the procedure the muscle was kept warm and moist by superfusion of warm buffered saline. In the applied in vivo model, leukocyte adhesion in cremaster muscle venules is induced by the surgical preparation and observed for up to 45 minutes after exteriorization of the cremaster muscle. Intravital microscopy was performed using a Zeiss Axiotech Vario microscope (Zeiss, Germany) and leukocyte-endothelium-interaction was analyzed in 5–6 postcapillary venules and recorded using a digital camera (AxioCam HSm, Zeiss Germany). For measurement of blood flow velocity, green fluorescent microspheres (1 µm diameter; Polysciences, Germany) were injected via the carotid artery catheter, and their passage through the vessels of interest was recorded using the respective fluorescent filter cube. Blood flow was calculated from the length of a single microsphere in a single image with defined exposure time. Rolling leukocytes were defined as those moving slower than the blood flow and their velocity was calculated from the distance they were moving in a defined time frame. Firmly adherent cells were determined as those resting in vessel for more than 30 seconds of observation and related to the luminal surface of the observed vessel part. For investigation of extravasated leukocytes, whole mounts of the Cremaster muscles were performed immediately after sacrifying the animals and Giemsa-staining was used to label perivascular leukocytes.

### Quantitative reverse transcriptase-polymerase chain reaction (RT-PCR) analysis

RT-PCR analysis was done as described [8]. Sequences were used as indicated or pre-developed Taq Man assay reagents or primers and probes were purchased from Applied Biosystems: NM_003265.2, HS00152933_m1 (human TLR3), NM_014314.3, Hs00204833_m1 (human RIG-I), NM_022168.2, Hs0170332_m1 (human MDA5), NM_000600.3, Hs00174131_m1 (human IL-6), NM_000584.2, Hs00174103_m1 (human IL-8), NM_002985.2, Hs00174575_m1 (human RANTES), NM_002982.3, Hs00234140_m1, (human MCP-1), NM_001565.2, Hs00171042_m1 (human IP-10), FP: CCT TCC TCC TGT CTG ATG GA; RP: ACT GGT TGC CAT CAA ACT CC; T1: 6FAM CAG ACA TGA CTT TGG ATT TCC CCA GG (human IFN-α), NM_002176.2, Hs00277188_s1 (human IFN-β), NM_172210.2, NM_172211.2, NM_172212.2, NM_000757.4 (human MCSF), NM_000201.2, Hs00164932_m1, (human ICAM-1), NM_001078.3, NM_001199834.1, NM_080682.2, Hs00365486_m1 (human VCAM-1), NM_000594.2, Hs00174128_m1 (human TNFα), NM_001065.3, Hs00533560_m1 (human TNFR1), NM_001066.2, Hs00153550_m1 (human TNFR2) and M33197 (human GAPDH).

### ELISA

ELISA for IL-6, IL-8 and IP-10 were performed on cell culture supernatants using commercial assay kits (R&D Systems, USA) and following providers instructions.

### Flow Cytometry

ICAM-1, VCAM-1, E- and P-Selectin located to the cell surface were measured after fixation with 4% formaldehyde and staining with anti-ICAM-1-FITC, anti-VCAM-1-FITC, anti-p-selectin-RPE and anti-Tissue-factor-FITC or the corresponding FITC- or RPE-labeled negative controls by a FACSCanto II flow cytometer (Becton Dickinson, USA). Data were analyzed using FACSDiva software (Becton Dickinson, USA). Antibodies and respective negative controls were purchased from Biozol (Germany), Southern Biotech (USA) or R&D Systems (USA) respectively.

### Western blot

Western blot analysis was performed as previously described [19]. HMEC were grown to subconfluence and starved for 24 hours in cell medium containing 1% FCS prior to stimulation with poly (I:C) for the indicated time intervals. After washing with PBS the cells were lysed on ice using cell lysis buffer (Cell Signaling Technology, USA) and protein concentration was determined using BCA (bicinchoninic acid) protein assay reagent kit according to the manufacturer's protocol. Equal amounts of protein were separated by gel electrophoresis (SDS-PAGE) and blotted onto a nitrocellulose membrane. Membranes were blocked by incubation 5% (w/v) BSA in TBSt (Tris-buffered saline with Tween) for 30 minutes prior to incubation with a rabbit anti-human-TNFR2-antibody (Cell signaling Technology, USA) at 4°C overnight. After washing with TBSt the membrane was incubated with a horseradish peroxidase conjugated secondary antibody for 1 hour at room temperature. Enzymatic activity was detected with a chemiluminescence detection kit according to the supplier's protocol and recorded with a digital camera (Hamamatsu). GAPDH served as loading control. Densiometric analysis of the blots was performed digitally using WASABI Software.

### Gene expression *in vivo*

Animal experiments were performed in wildtype (WT) or TNFα receptor subtype 2 deficient C57BL/6 mice. For gene expression studies in vivo, poly (I:C) or sham treated mice were sacrified and lungs, kidneys, aorta and cremaster muscle were immediately isolated and homogenized by adding ceramic microspheres to the tissue and mixing with a CapMix (3 M ESPE, Germany). RNA isolation was then performed using a commercially available RNA isolation kit (PeqLab, Germany). Samples were kept frozen at −20°C until further analysis. For real-time PCR, commercially available pre-developed TaqMan reagents were used for the mouse target genes TLR3 and TNFR2. GAPDH was used as reference housekeeping gene. All measurements were performed in duplicates.

### Knockdown of gene expression with short interfering RNA (siRNA)

Predesigned siRNAs specific for TLR3, RIG-I and MDA5 were purchased from Ambion (Japan). Transfection of cells with siRNA was performed as described before [13]. Scrambled siRNA was used as the nonspecific negative control of siRNA.

### Proliferation assays

To assess the proliferative activity of HMEC, MTT assays were performed as described [20]. Aliquots of 5×10^3^ cells in 100 µl culture medium were cultured in 96-well microtiter plates for 24 hours under standard conditions to yield firmly attached and stably growing cells. After discarding the supernatants, cells were incubated with poly (I:C) as indicated. After removal of the supernatant, 50 µl of a 1 mg/ml solution of MTT (Sigma-Aldrich, USA) were added for 3 hours at 37°C. Then, formazan crystals were dissolved by the addition of 50 µl isopropanol. Absorbance was measured at 570 to 690 nm as a reference using a Dynatech MR7000 ELISA reader (Denkendorf, Germany).

### Preparation of HCV RNA

HCV RNA containing cryoprecipitates were isolated from a patient with a HCV associated MC with a high viral load during routine plasmapheresis treatment and centrifuged. as described previously [10] The concentration of HCV used for stimulation was 100 or 200×10^6^ geq/ml, confirmed by RT-PCR. For HCV stimulation, confluent HMEC in 6-well plates were used; once the virus was added, the plates were centrifuged at 1000 g for 45 min to allow efficient viral infection. Subsequent stimulation was performed as indicated.

### Histology

Immediately after sacrificing the animals organs were isolated, removed from the surrounding connective tissue and stored in paraformaldehyde until further analysis [21].

### Statistical analysis

Values are provided as mean ± SD. Statistical analysis was performed by the unpaired t test if applicable or by the ANOVA-analysis. Significant differences in expression levels are indicated for p values <0.05 (_*_) or 0.01 (_**_), respectively.

## Results

### Expression of viral RNA receptors of the innate immune system on human microvascular endothelial cells (HMEC)

**Figure 1:**
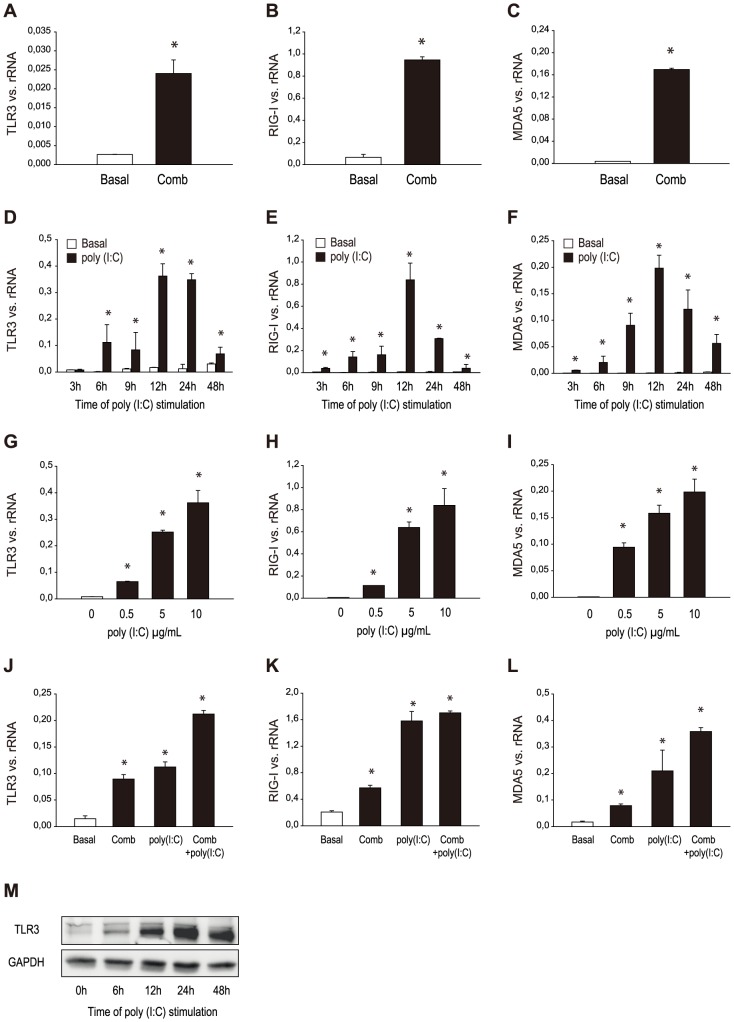
Expression of viral receptors in HMEC. HMEC were stimulated with (Comb) and without (Basal) a combination of the proinflammatory cytokines TNFα, IL-1β and IFN-γ for 12 hours. By RT-PCR, specific products for TLR3 (A), RIG-I (B) and MDA5 (C) mRNA were amplified from both unstimulated and stimulated cells. HMEC were stimulated without and with poly (I:C) (10 µg/ml) for different time intervals (3, 6, 9, 12, 24, 48 hours) and mRNA expression of TLR3 (D), RIG-I (E) and MDA5 (F) was analyzed by RT-PCR. Time-dependent effect of poly (I:C) on TLR3 protein expression was confirmed by western blot (M). HMEC were stimulated without (Basal) and with different concentrations (0.5, 5, 10 µg/ml) of poly (I:C) for 12 hours and expression of TLR3 (G), RIG-I (H) and MDA5 (I) was measured by RT-PCR. HMEC were pretreated under basal or cytokine combination conditions (Comb) as described in Methods and after washing incubated with or without poly (I:C) (10 µg/ml) for 12 hours. Expression of TLR3 (J), RIG-I (K) and MDA5 (L) was analyzed by RT-PCR. Results are given as means ± SD of three experiments done in parallel for each condition and rRNA served as the reference gene. Comparable results were obtained in two series of independent experiments. Statistically significant differences to the control are depicted with *  = p≤0.05.

We examined cultured HMEC for the expression of the viral dsRNA sensing receptors TLR3, RIG-I and MDA5. RNA was prepared from cells grown under standard conditions as well as from cells that had been stimulated with a combination of the cytokines TNFα, IL-1β and IFN-γ for 12 hours to simulate a proinflammatory milieu as would occur during immune-mediated vasculitis. By RT-PCR, specific products for TLR3, RIG-I and MDA5 mRNA were amplified from both unstimulated and stimulated cells. The basal expression for TLR3, RIG-I and MDA5 was induced by the cytokine combination (Figure 1A–C). In order to test the effect of ligand binding on the expression of viral receptors, HMEC were stimulated with poly (I:C) (10 µg/ml) mimicking viral RNA for different time intervals (3, 6, 9, 12, 24, 48 hours). Poly (I:C) led to a time dependent increase in the expression of viral receptors with a maximum after 12 hours (Figure 1D–F). The time-dependent effect of poly (I:C) on TLR3 protein synthesis was demonstrated by western blot (Figure 1M). Stimulation of HMEC with different concentrations (0.5, 5, 10 µg/ml) of poly (I:C) for 12 hours led to a concentration-dependent increase in the expression of TLR3, RIG-I and MDA5 (Figure 1G-I). Furthermore, HMEC were cultivated under basal conditions or pretreated with a combination of the proinflammatory cytokines TNF-α, IL-1β and IFN-γ as described, washed and then incubated with poly (I:C) (10 µg/ml) for 12 hours. mRNA levels of TLR3, RIG-I and MDA5 were measured. Again, the cytokine combination led to an enhanced expression of the viral receptors TLR3, RIG-I and MDA5; the effect of poly (I:C) on the expression of TLR3 and MDA5 was amplified in cells pretreated with proinflammatory cytokines. (Figure 1J–L). Additionally, HMEC were screened for mRNA expression of TLR1 through TLR10 by RT-PCR under basal conditions and after cytokine pretreatment. HMEC show a robust expression of TLR1, TLR4 and TLR6 without significant changes after stimulation with proinflammatory cytokines. Under basal conditions, no relevant expression was found for TLR2 and TLR7, but these receptors were inducible by the cytokine combination. The expression for TLR9, both basal and after stimulation, was too low to allow for evaluation. No expression was found for TLR5, TLR8 and TLR10 under any condition (results not shown).

### Effect of poly (I:C) stimulation on the expression of selected cytokines and chemokines, type I interferons, MCSF and adhesion molecules

HMEC were stimulated with poly (I:C) (10 µg/ml) for different time intervals (3, 6, 9, 12, 24, 48 hours) and the expression of selected proinflammatory cytokines and chemokines was analyzed by RT-PCR. Poly (I:C) increased the mRNA expression of IL-6, IL-8, IP-10, RANTES and MCP-1 in a time dependent manner with a maximum after 12 hours of stimulation time (Figure S1A, C, E, G, H). On protein level, this effect was confirmed for IL-6, IL-8 and IP-10 by ELISA (Figure S1B, D, F).

In contrast to the basal expression of IFN-α on HMEC, which was not significantly changed by incubation with poly (I:C), the expression of IFN-β was early and stably increased from 3 up to 12 hours of poly (I:C) stimulation. (Figure S1I, J). MCSF expression was elevated over the whole poly (I:C) stimulation interval from 3 to 48 hours, with a maximum after 12 hours stimulation (Figure S1K). mRNA expression of ICAM-1 and VCAM-1 were increased from 3 to 24 hours poly (I:C) stimulation, also with a maximum after 12 hours (figure S1L, N); a concordant increase in protein synthesis of these adhesion molecules was confirmed by FACS (Figure S1M, O). Additionally, the surface expression of E-selectin and P-selectin was analyzed in human umbilical vein endothelial cells (HUVEC) by FACS. When HUVEC were stimulated with poly (I:C) for 24 hours, P-selectin expression was significantly increased, whereas the expression of E-selectin was increased from 24 to 48 hours of poly (I:C) treatment (Figure S1P, Q). The stimulating effect of poly (I:C) was also reproducible in HUVEC for the selected targets IL-6 and ICAM-1 (data not shown).

### Effect of poly (I:C) on endothelial cell proliferation

Proliferation of HMEC in response to poly (I:C) was assessed by the MTT proliferation assay as described. When HMEC were stimulated with poly (I:C) (10 µg/ml) for different time intervals (3, 6, 9, 12, 24, 48 hours), cell proliferation was significantly decreased from 6 up to 48 hours stimulation time (Figure S1R).

### Effect of HCV-RNA containing cryoprecipitates on the expression of cytokines, chemokines and adhesion molecules

**Figure 2:**
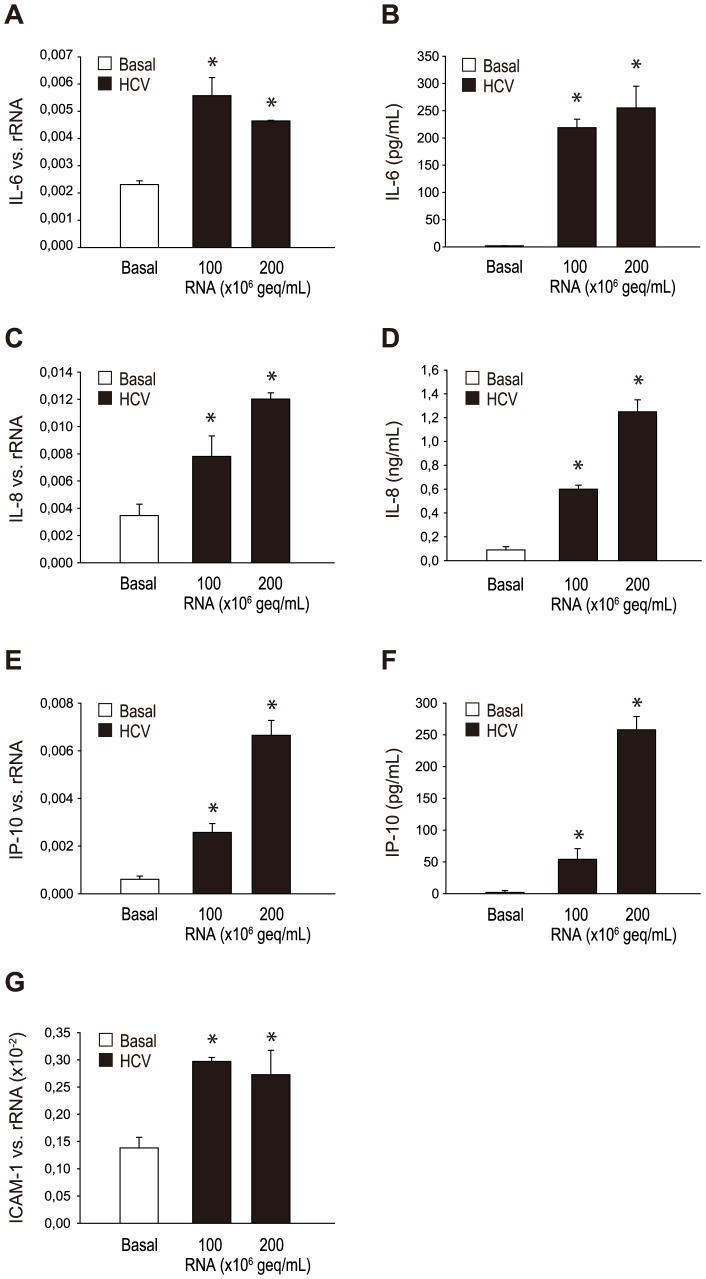
Effect of HCV RNA containing cryoprecipitates on the expression of cytokines, chemokines and adhesion molecules. HMEC were stimulated with different concentrations (100×10^6^ geq/ml, 200×10^6^ geq/ml) of HCV RNA containing cryoprecipitates (HCV) for 12 hours and mRNA expression of selected cytokines and chemokines IL-6 (A), IL-8 (C), IP-10 (E) and adhesion molecule ICAM-1 (G) was analyzed by RT-PCR. Protein synthesis of IL-6 (B), IL-8 (D) and IP-10 (F) was also confirmed by ELISA. Results are given as means ± SD of three experiments done in parallel for each condition and rRNA served as the reference gene. Comparable results were obtained in two series of independent experiments. Statistically significant differences to the control are depicted with *  = p≤0.05.

As we hypothesize a role of receptors of the innate immune system in Hepatitis C associated vascular inflammation and as we could demonstrate a role of TLR3 in the mediation of endothelial inflammation and production of adhesion molecules, we next tested the effect of a stimulation with HCV-RNA containing cryoprecipitates from a patient with an Hepatitis C-associated cryoglobulinemia on the expression of selected cytokines, chemokines and adhesion molecules. Cryoprecipitates were isolated as described in Materials and Methods and HMEC were stimulated with different concentrations (100×10^6^ geq/ml, 200×10^6^ geq/ml) of HCV RNA for 12 hours. HCV RNA containing cryoprecipitates increased the mRNA expression of IL-6, IL-8 and ICAM-1 (Figure 2A, C, E, G). A concordant increase in protein synthesis was confirmed by ELISA for IL-6, IL-8 and IP-10 (Figure 2B, D, F).

### Effect of transfection with siRNA specific for TLR3, RIG-I and MDA5 on poly (I:C) induced gene expression

To define the viral receptor which mediates the poly (I:C)-dependent induction of the dsDNS sensing receptors, HMEC were transfected with siRNA specific for TLR3, RIG-I and MDA5 as described and stimulated with poly (I:C) (10 µg/ml) for 12 hours. Expression of TLR3, RIG-I and MDA5 was measured by RT-PCR. The poly (I:C)-dependent induction of the expression of TLR3 was significantly blocked only by siRNA specific for TLR3; the poly(I:C)-dependent induction of RIG-I was reduced by transfection with siRNA specific for TLR3 and RIG-I, and the expression of MDA5 was reduced by the knockdown of any of the three RNA receptors (Figure S2A–C). To identify the viral receptor responsible for the induction of cytokines, chemokines and adhesion molecules by poly (I:C), HMEC were transfected with siRNA specific for TLR3, RIG-I and MDA5 and stimulated with poly (I:C) (10 µg/ml) for 12 hours. Poly (I:C) increased the expression of the selected proinflammatory genes IL-6, RANTES, MCP-1, IP-10, IFN-β and VCAM-1, and these effects were significantly reduced by transfection of HMEC with siRNA specific for TLR3; transfection with siRNA specific for RIG-I, MDA5 and with negative controls containing unspecific RNA had no effect (Figure S2D-I).

### Effect of poly (I:C) on vascular inflammation *in vivo*

**Figure 3:**
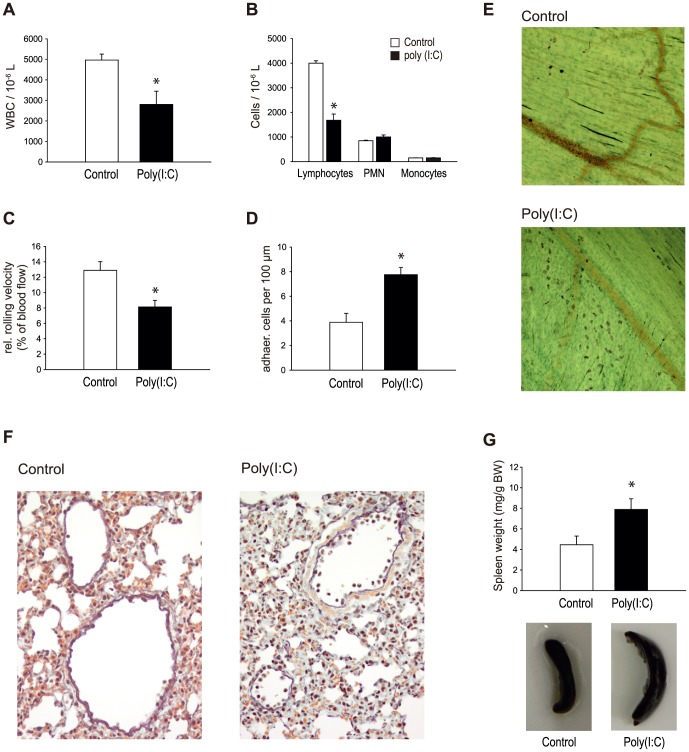
Effect of poly (I:C) on vascular inflammation in vivo. C57Bl/6 mice were systemically treated with poly (I:C) (200 µg i.p.) and white blood cell counts were perfomed after 48 hours (A, B). At the same time leukocyte rolling (C) and adhesion (D) in vivo as well as extravasation of leukocytes (E) were investigated in postcapillary venules of the mouse cremaster muscle by intravital microscopy or in cremaster muscle whole mounts respectively. After repeated ploly (I:C) treatment (100 µg every other day for 2 weeks) vascular inflammation was investigated in lung tissue as described (F) and spleen weight was measured (G). Results are given as means ± SEM (n = 4–6 animals). Statistically significant differences to the control are depicted with *  = p≤0.05.

To test whether poly (I:C)-induced upregulation of inflammatory cytokines and adhesion molecules on endothelial cells in vitro translates into vascular inflammation in vivo, C57Bl/6 mice were systemically treated with poly (I:C) (200 µg i.p.). After 48 h, the white blood cell count was significantly decreased compared to controls, which was due to a decrease in lymphocyte counts (Figure 3A, B). Leukocyte rolling and adhesion were observed by intravital microscopy in trauma-stimulated cremaster muscle venules. While hemodynamic and microvascular parameters were similar to sham treated controls, leukocyte rolling velocity was significantly slower in the poly (I:C) treated group (Figure 3C). Additionally, poly (I:C) increased the fraction of leukocytes adhering to the vessel wall and consecutively led to a significantly increased extravasation of leukocytes as detected in whole mounts of cremaster muscles after sacrificing the animals (Figure 3D, E). Repeated treatment of animals with poly (I:C) (100 µg every other day for 2 weeks) resulted in a significantly increased leukocyte adherence in the lung tissue (Figure 3F) as well as in prominent splenomegaly (Figure 3G).

### Effect of TNFa on the expression of cytokines, chemokines, MCSF and adhesion molecules

As we have previously demonstrated a role for TNFα and its receptors in hepatitis C virus-associated glomerulonephritis and vascular thrombosis [10], [22] and as we therefore hypothesize a predominant role of the TNFα/TNF receptor system in vascular inflammation, we now tested the effect of TNFα on the expression of selected proinflammatory cytokines, chemokines, MCSF and adhesion molecules. Stimulation of HMEC with TNFα for different time intervals (3, 6, 9, 12, 24 hours) led to a significant increase in the expression of IL-6, IL-8, IP-10 RANTES, MCP-1, IP-10, IFN-β, MCSF, ICAM-1 and VCAM-1 from 3 to 24 hours, with a maximum after a 3 hours' stimulation time for IL-6 and IL-8, after 12 hours for IP-10 and RANTES after 24 hours for IFN-β (figure S3A, C, E, G, I). mRNA expression of MCP-1, MCSF, ICAM-1 and VCAM-1 was stably increased from 3 to 24 hours of TNFα stimulation (Figure S3H, J, K, L). Increased protein levels of IL-6, IL-8 and IP-10 (Figure S3B, D, F) and ICAM-1 (data not shown) were confirmed by ELISA or FACS.

### Effect of poly (I:C) stimulation and HCV RNA containing cryoprecipitates on the expression of TNFα and TNF receptors

**Figure 4:**
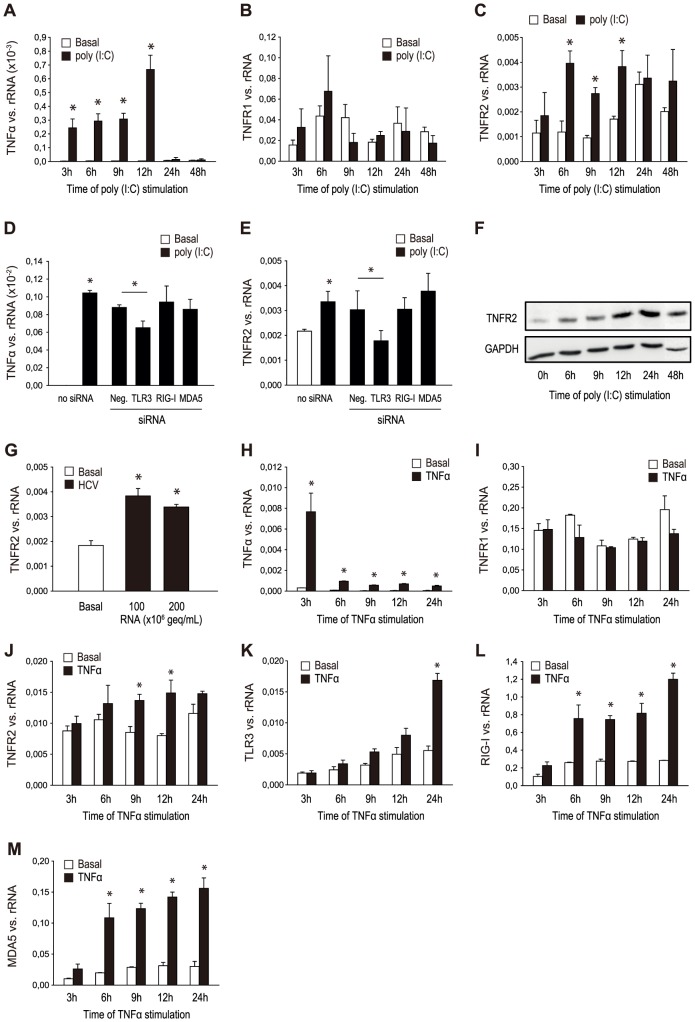
Effect of poly (I:C) and HCV RNA containing cryoprecipitates on the expression of TNFα and TNF receptors; effect of TNFα on the expression of TNF receptors and viral receptors. HMEC were stimulated without (basal) and with poly (I:C) (10 µg/ml) for different time intervals (3, 6, 9, 12, 24, 48 hours) and expression of TNFα (A), TNF receptor 1 (TNFR1) (B) and TNF receptor 2 (TNFR2) (C) was analyzed by RT-PCR. (D, E) HMEC were transfected with siRNA for TLR3, RIG-I and MDA5 as well as unspecific RNA as negative control as described in Materials and Methods and stimulated with poly (I:C) (10 µg/ml) for 12 hours. Expression of TNFα (D) and TNFR2 (E) was analyzed by RT-PCR. Protein expression of TNFR2 after different time intervals of poly (I:C) stimulation (3, 6, 9, 12, 48 hours) was confirmed by western blot (F). HMEC were stimulated with different concentrations (100×10^6^ geq/ml, 200×10^6^ geq/ml) of HCV RNA containing cryoprecipitates for 12 hours and mRNA expression of TNFR2 (G) was analyzed by RT-PCR. HMEC were stimulated with TNFα for different time intervals (3, 6, 9, 12, 24 hours) and expression of TNFα (H), TNFR1 (I), TNFR2 (J), TLR3 (K), RIG-I (L) and MDA5 (M) was analyzed by RT-PCR. Results are given as means ± SD of three experiments done in parallel for each condition and rRNA served as the reference gene. Comparable results were obtained in two series of independent experiments. Statistically significant differences to the control are depicted with *  = p≤0.05.

Still based on the hypothesis of a predominant role of the TNFα/TNF receptor system in immune-mediated vascular inflammation, we then tested the effect of activation of viral receptors on the expression of TNFα and TNF receptors in HMEC. HMEC were stimulated with poly (I:C) (10 µg/ml) for different time intervals (3, 6, 9, 12, 24, 48 hours) and the expression of TNFα and the TNF receptor subtypes TNF receptor 1 (TNFR1) and TNF receptor 2 (TNFR2) was analyzed by RT-PCR. Poly (I:C) stimulation increased the expression of TNFα from 3 to 12 hours of poly (I:C) stimulation time, with a maximum after 12 hours. Knockdown experiments with siRNA specific for TLR3, RIG-I and MDA5 showed this effect to be TLR3-dependent (Figure 4A, D). Poly (I:C) stimulation showed a tendency to early enhance the expression of TNFR1, which though did not reach statistical significance (Figure 4B). TNFR2 mRNA expression was significantly increased after 6, 9 and 12 hours of poly (I:C) stimulation (Figure 4C), an effect which was also shown to be TLR3-dependent and confirmed on protein level by western blot (Figure 4E, F). The stimulating effect of poly (I:C) on TNFR2 was reproducible in HUVEC (data not shown). Furthermore, stimulation with HCV-RNA containing cryoprecipitates in different concentrations (100×10^6^ geq/ml, 200×10^6^ geq/ml), which had been isolated from a patient with HCV-associated cryoglobulinemia, for 12 hours led to a significant increase in the expression of TNFR2 (Figure 4G).

### Effect of TNFα on the expression of TNF receptors and viral receptors

To test for an amplification of proinflammatory responses upon activation of viral receptors by TNFα itself, HMEC were stimulated with TNFα for different time intervals (3, 6, 9, 12, 24 hours) and the expression of TNFα, the TNF receptors TNFR1 and TNFR2 as well as of the viral receptors TLR3, RIG-I and MDA5 was analyzed by RT-PCR. TNFα led to an increase in the expression of TNFα and TNFR2 from 3 to 24 hours respectively 9 to 12 hours of stimulation time, but had no effect on the basal expression of TNFR1 (Figure 4H–J). Expression levels of TLR3 were significantly increased only after a 24 hours' stimulation with TNFα, whereas the expression of RIG-I and MDA5 was already increased after 6 hours and reached a maximum after 24 hours of TNFα stimulation (Figure 4K-M).

### Amplification of poly (I:C) dependent induction of viral receptors and TNF receptors 2 by TNFα

To test for an additionally permissive effect of TNFα on the ligand dependent induction of the dsRNA sensing viral receptors TLR3, RIG-I and MDA5 as well as on TNFR2, HMEC were incubated with poly (I:C) (10 µg/ml) for 24 hours and then incubated in culture medium or stimulated with TNFα for different time intervals (6, 12, 24 hours). As expected, the expression of TLR3, RIG-I and MDA5 was increased by poly (I:C), and the subsequent incubation with TNFα further induced expression levels of all these viral receptors and of TNFR2 (Figure S4A–D).

### Amplification of poly (I:C) dependent induction of cytokines, chemokines and adhesion molecules by TNFα

**Figure 5:**
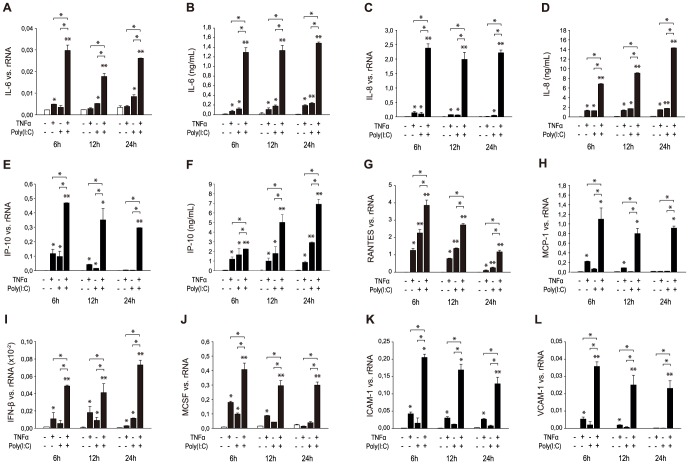
Amplification of poly (I:C)-mediated induction of cytokines, chemokines and adhesion molecules by TNFα. HMEC were grown in culture medium for 24 hours and additionally incubated in medium (basal) for different time intervals (6, 12, 24 hours) or grown in culture medium for 24 hours and additionally incubated with TNFα (TNFα) for different time intervals (6, 12, 24 hours) or incubated with poly (I:C) for 24 hours and then grown in medium (poly (I:C)) or stimulated with TNFα (poly (I:C) + TNFα) for different time intervals (6, 12, 24 hours). mRNA expression of selected cytokines and chemokines IL-6 (A), IL-8 (C), IP-10 (E), RANTES (G), MCP-1 (H), IFN-β (I), MCSF (J), ICAM-1 (K) and VCAM-1 (L) was analyzed by RT-PCR. Protein synthesis of selected targets IL-6 (B), IL-8 (D) and IP-10 (F) was also confirmed by ELISA. Results are given as means ± SD of three experiments done in parallel for each condition and rRNA served as the reference gene. Comparable results were obtained in two series of independent experiments. Statistically significant differences to the control are depicted with * = p≤0.05, ** = p≤0.01.

To test whether TNFα would also directly amplify endothelial inflammatory responses seen upon activation of viral receptors by poly (I:C), HMEC were stimulated by poly (I:C) (10 µg/ml) for 24 hours and subsequently incubated with TNFα for different time intervals (6, 12, 24 hours). Poly (I:C) led to an increase in the expression of IL-6, IL-8, IP-10, RANTES, IFN-β and MCSF (Figure 5A, C, E, G, I, J), whereas MCP-1, ICAM-1 and VCAM-1 were not significantly increased by stimulation with poly (I:C) alone (figure 5H, K, L). The stimulatory effects of poly (I:C) were significantly potentiated by TNFα treatment for 6, 12 and 24 hours. A parallel increase in protein synthesis of IL-6, IL-8 and IP-10 was confirmed by ELISA (Figure 5B, D, H).

### Role of TNFR2 in poly (I:C) induced vascular inflammation

**Figure 6:**
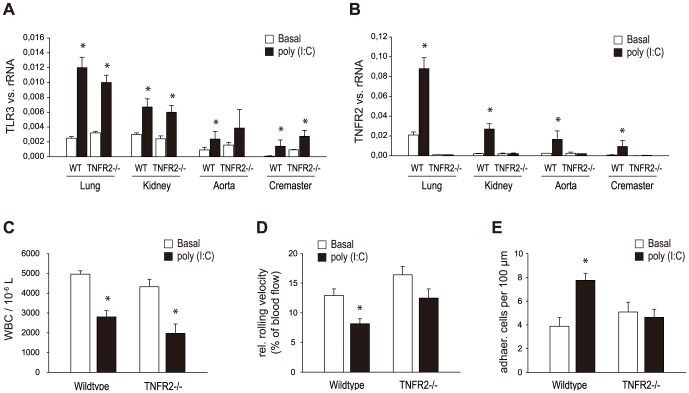
Role of TNFR2 in poly (I:C) induced vascular inflammation. WT and TNFR2^-/-^ mice were sham treated or treated with poly (I:C) 200 µg i.p. as described and expression of TLR3 and TNFR2 was analyzed by RT-PCR. Expression of TLR3 (A) and TNFR2 (B) was measured in lung, kidney, aorta and cremaster muscle tissue after 48 hours. At the same time point white blood cell counts were performed and effects of poly (I:C) on trauma-induced leukocyte rolling and adherence in vivo were investigated in cremaster muscle venules by intravital microscopy (C–E). Statistically significant differences to the control are depicted with *  = p≤0.05.

We next investigated the expression of TLR3 and TNFR2 upon activation of viral RNA receptors in organ lysates of wild-type (WT) and TNFα receptor subtype 2-deficient (TNFR2^-/-^) C57BL/6 mice. WT or TNFR2^-/-^ mice were treated with poly (I:C) 200 µg i.p. as described and TLR3 and TNFR2 mRNA levels were analyzed after 24 and 48 hours. After 24 hours, poly (I:C) had no effect on TLR3 or TNFR2 expression in lung tissue of WT or TNFR2^-/-^ mice (data not shown), whereas after 48 hours poly (I:C) upregulated TLR3 expression in lung, kidney, aorta and cremaster muscle of both WT and TNFR2^-/-^ mice (Figure 6A). Moreover, after 48 hours of poly (I:C) treatment, a significant increase in the expression of TNFR2 in lung, kidney, aorta and cremaster muscle of WT mice was observed; obviously, no expression of TNFR2 was found in TNFR2^-/-^ mice (Figure 6B). Poly (I:C) decreased the white blood cell count in both WT and TNFR2^-/-^ mice (Figure 6C). The poly (I:C)-dependent increase in rolling leukocytes in cremaster muscle venules and in leukocyte adherence in vivo reached statistical significance only in WT, not in TNFR2-/- mice (Figure 6D, E).

## Discussion

Hepatitis C virus (HCV)-related systemic vasculitis is a severe complication of infection with HCV and causes significant morbidity and mortality [23]. HCV-associated vasculitis has been described to occur both in the presence and in the absence of mixed cryoglobulinemia (MC) [24], and up to 10% of patients with MC develop systemic vasculitis [6], [7]. From the patients found to have MC, more than 80% are infected by HCV [2], [3], [5], [6]. During disease course, immune complexes containing viral RNA reach the small and medium-sized arteries and veins and deposit in the vessel walls. The clinical features of HCV-associated vasculitis not only include the classical triad of purpura, arthralgias and myalgias, but also prognostically severe forms of glomerulonephritis, lymphoproliferative disorders, neuropathies and skin ulcerations as well as other dermatologic conditions [25].

Cytokines and chemokines play a central role in the immune response to HCV as they mediate antiviral and proinflammatory effects in immune as well as primarily non-immune cell types [26], [27], [28]. In the clinical setting, serum levels of proinflammatory mediators including IL-6 and TNFα have been shown to be increased in patients with an HCV-associated MC and are found to be particularly elevated during active vasculitis [29]. Furthermore, the emergence of severe vasculitis due to HCV-associated MC was shown to be dependent on the induction of adhesion molecules including VCAM-1 and ICAM-1, both involved in mononuclear respectively polymorphonuclear and mononuclear cell recruitment during vasculitis [30]. However, so far there are no data on the contribution of endothelial viral receptors of the innate immune system, which are able to bind nucleic acids of viral origin and elicit proinflammatory responses, to HCV-associated vasculitis. There is large body of evidence for several receptors for dsRNA existing in eukaryontic cells, including TLR3 and the cytosolic receptors RIG-I and MDA5 [31], [32]. In human and murine endothelial cells, the synthetic analogue of dsRNA of viral origin, poly (I:C), is known to have various proinflammatory effects [33], [34], [35]. These effects depend on the origin of the endothelial cells as to species and vascular bed [36].

In the present manuscript we demonstrate for the first time that HCV-RNA isolated from a patient with HCV-associated MC significantly induces both expression and synthesis of a variety of mediators known to be relevant to the initiation and propagation of inflammation in viral disease, and the adhesion molecule ICAM-1. We infer that HCV directly promotes activation, adhesion and infiltration of inflammatory cells into the vessel wall by activation of viral receptors of the innate immune system in endothelial cells. Due to the ability of HCV to both recruit leucocytes to the endothelium and sustain local inflammatory responses, any relevant viremia could cause a clinically apparent vasculitis. As a matter of fact, in mice systemically treated with the synthetic analogue of viral dsRNA poly (I:C), we found a significant reduction in leukocyte rolling velocity as well as an increase in leukocyte adhesion to the vessel wall, which was paralleled by a significantly increased extravasation of leukocytes in vivo. In addition, poly (I:C) induces the expression of the corresponding receptor TLR3 in vivo and hereby allows for amplification of all of the aforementioned responses upon viral infection. These findings underscore the relevance of direct effects of circulating nucleic acids of viral origin on the pathogenesis of vasculitis. In vitro stimulation of viral receptors with poly (I:C) causes a time- and dose- dependent induction of IL-6, IL-8, RANTES, MCP-1, IP-10 and IFN-β as well as MCSF and the adhesion molecules ICAM-1 and VCAM-1 in HMEC. Using knockdown experiments with siRNAs specific for the viral receptors TLR3, RIG-I and MDA5, we provide evidence for a mutual dependence of the regulation of viral RNA receptors upon ligand binding as well as for a selective mediation of the poly (I:C)-dependent induction of proinflammatory genes by TLR3. We presume these effects to promote vascular inflammation in vivo once circulating HCV-RNA binds to viral receptors of the innate immune system, a process which does not presuppose viral entry and replication in endothelial cells. Even though not classifying as primary target cells of HCV, however, HMEC express CD81 tetraspanin, the scavenger receptor class B type I, tight junction proteins and LDL receptors, all of them able to mediate HCV entry into hepatic and non-hepatic cells [37], [38]. Furthermore, human brain endothelial cells have already been found to express functional receptors that support HCV entry and replication [39]. The contribution of a direct viral infection of HMEC to extrahepatic manifestations of HCV infection thus merits future attention.

An important mediator involved in both the early antiviral response and a variety of immune and autoimmune phenomena associated with viral diseases is tumor necrosis factor alpha (TNFα). Referring to vasculitis in particular, TNFα is known to partly mediate the prothrombotic conditions implicated by systemic inflammation, and small vessel occlusion results in an amplification of local inflammatory responses. In this manuscript we show that TNFα adds significantly to the proinflammatory effects of viral RNA on endothelial cells and therefore plays an important role in the initiation and propagation of vascular manifestations of HCV-infection. In addition, we show for the first time that HCV-RNA containing cryoprecipitates significantly induce the endothelial expression of TNFα and TNFα receptor subtype 2 (TNFR2) in vitro and we provide evidence that leucocyte adhesion and transmigration in response to activation of viral RNA receptors in vivo seem to depend on the expression of functional TNFR2. While TNFR1 is constitutively expressed in most tissues, the expression of TNFR2 is highly regulated and mediates tissue damage in chronic inflammation; this effect possibly depends on the induction of endogenous TNFα via activation of TNFR2, which increases local TNFα concentrations and subsequently activates TNFR1 [40]. Our observation of a selective induction of TNFR2 by HCV-RNA and the requirement of functional TNFR2 for endothelium-dependent leucocyte activation in poly (I:C)-treated mice would again favour the therapeutic use of a TNFR2-specific antagonist, already advocated for its potential to downregulate excessive TNFα signaling in uncontrolled inflammatory processes without affecting beneficial TNFα effects. In view of the fact that the treatment of HCV-associated vasculitis is largely empirical, this alternative therapeutic approach might be of particular clinical relevance. Indeed, the current standard therapy is antiviral treatment with peg-inteferon plus ribavirin without use of systemic immunosuppressive agents [6], [41]; for MC, a therapy with Rituximab, a monoclonal antibody against the CD 20 antigen expressed on B cells, can be considered in patients with severe clinical manifestations including vasculitis, glomerulonephritis or neuropathy [42].

In summary, our results demonstrate that endothelial cells actively participate in immune mediated vascular inflammation caused by viral infections. Activation of the pattern-recognition receptor TLR3 by HCV-RNA and poly (I:C) induces the expression of proinflammatory cytokines, chemokines and adhesion molecules and might thereby directly contribute to viral disease-associated vasculitis. TNFα adds significantly to the proinflammatory effects of viral RNA on endothelial cells. In vivo, poly (I:C) enhances leucocyte adhesion and transmigration, an effect which seems to selectively depend on subtype specific signalling of TNFα via TNFR2.

## Supporting Information

Figure S1 **Effect of poly (I:C) on the expression of cytokines, chemokines, type I interferons, MCSF and adhesion molecules on HMEC.** HMEC were stimulated without (basal) and with poly (I:C) (10 µg/ml) for different time intervals (3, 6, 9, 12, 24, 48 hours) and mRNA expression of IL-6 (A), IL-8 (C), IP-10 (E), RANTES (G), MCP-1 (H), IFN-α (I), IFN-β (J), MCSF (K), ICAM-1 (L) and VCAM-1 (N) was analyzed by RT-PCR. Protein synthesis of selected factors IL-6 (B), IL-8 (D) and IP-10 (F) was confirmed by ELISA. Protein synthesis of ICAM-1 (M) and VCAM-1 (O) was confirmed by FACS. Surface expression of E-selectin (P) and P-selectin (Q) was analyzed in HUVEC by FACS. Results are given as means ± SD of three experiments done in parallel for each condition and rRNA served as the reference gene. Comparable results were obtained in two series of independent experiments. (R) HMEC were stimulated with poly (I:C) (10 µg/ml) for different time intervals (0, 3, 6, 9, 12, 24, 48 hours) and cell proliferation was analyzed by the MTT assay. Each bar represents a mean ± SD of 24 parallel incubations for each condition. Comparable results were obtained in four series of independent experiments. Statistically significant differences to the control are depicted with *  = p≤0.05.
(EPS)

Figure S2 **Effects of transfection with siRNA for TLR3, RIG-I and MDA5 on poly (I:C) induced gene expression.** HMEC were transfected with siRNA for TLR3, RIG-I and MDA5 as well as unspecific RNA as negative control as described in Materials and Methods and stimulated with poly (I:C) (10 µg/ml) for 12 hours. Expression of TLR3 (A), RIG-I (B), MDA5 (C) IL-6 (D), RANTES (E), MCP-1 (F), IP-10 (G), IFN-β (H), ICAM-1 (I) and VCAM-1 (J) was analyzed by RT-PCR. Results are given as means ± SD of three experiments done in parallel for each condition and rRNA served as the reference gene. Comparable results were obtained in two series of independent experiments. Statistically significant differences to the control are depicted with *  = p≤0.05.
(EPS)

Figure S3 **Effect of TNFα on the expression of cytokines, chemokines and adhesion molecules.** HMEC were stimulated with TNFα for different time intervals (3, 6, 9, 12, 24 hours) and mRNA expression of IL-6 (A), IL-8 (C), IP-10 (E), RANTES (G), MCP-1 (H), IFN-β (I), MCSF (J), ICAM-1 (K) and VCAM-1 (L) was analyzed by RT-PCR. Protein synthesis of IL-6 (B), IL-8 (D) and IP-10 (F) was analyzed by ELISA. Results are given as means ± SD of three experiments done in parallel for each condition and rRNA served as the reference gene. Comparable results were obtained in two series of independent experiments. Statistically significant differences to the control are depicted with *  = p≤0.05, **  = p≤0.01.
(EPS)

Figure S4 **Amplification of poly (I:C)-dependent induction of viral receptors and TNF receptor 2 by TNFα.** HMEC were grown in culture medium for 24 hours and additionally incubated in medium (basal) for different time intervals (6, 12, 24 hours) or grown in culture medium for 24 hours and additionally incubated with TNFα (TNFα) for different time intervals (6, 12, 24 hours) or incubated with poly (I:C) for 24 hours and then grown in medium (poly (I:C)) or stimulated with TNFα (poly (I:C) + TNFα) for different time intervals (6, 12, 24 hours). Expression of TLR3 (A), RIG-I (B) and MDA5 (C) as well as TNFR2 (D) was analyzed by RT-PCR. Results are given as means ± SD of three experiments done in parallel for each condition and rRNA served as the reference gene. Comparable results were obtained in two series of independent experiments. Statistically significant differences to the control are depicted with *  = p≤0.05.
(EPS)

## References

[1] Poynard T, Yuen MF, Ratziu V, Lai CL (2003) Viral hepatitis C. Lancet 362:2095–2100. 14697814 10.1016/s0140-6736(03)15109-4

[2] Agnello V, Chung RT, Kaplan LM (1992) A role for hepatitis C virus infection in type II cryoglobulinemia. N Engl J Med 327:1490–1495. 1383822 10.1056/NEJM199211193272104

[3] Ferri C, Greco F, Longombardo G, Palla P, Moretti A, et al (1991) Antibodies to hepatitis C virus in patients with mixed cryoglobulinemia. Arthritis Rheum 34:1606–1610. 1660716 10.1002/art.1780341221

[4] Perico N, Cattaneo D, Bikbov B, Remuzzi G (2009) Hepatitis C infection and chronic renal diseases. Clin J Am Soc Nephrol 4:207–220. 19129320 10.2215/CJN.03710708

[5] Cacoub P, Fabiani FL, Musset L, Perrin M, Frangeul L, et al (1994) Mixed cryoglobulinemia and hepatitis C virus. Am J Med 96:124–132. 7509124 10.1016/0002-9343(94)90132-5

[6] Cacoub P, Poynard T, Ghillani P, Charlotte F, Olivi M, et al (1999) Extrahepatic manifestations of chronic hepatitis C. MULTIVIRC Group. Multidepartment Virus C Arthritis Rheum 42:2204–2212. 10.1002/1529-0131(199910)42:10<2204::AID-ANR24>3.0.CO;2-D 10524695

[7] Vassilopoulos D, Calabrese LH (2002) Hepatitis C virus infection and vasculitis: implications of antiviral and immunosuppressive therapies. Arthritis Rheum 46:585–597. 11920393 10.1002/art.10107

[8] Wörnle M, Schmid H, Banas B, Merkle M, Henger A, et al (2006) Novel role of toll-like receptor 3 in hepatitis C-associated glomerulonephritis. Am J Pathol 168:370–385. 16436653 10.2353/ajpath.2006.050491 PMC1606499

[9] Wörnle M, Roeder M, Sauter M, Ribeiro A (2009) Role of matrix metalloproteinases in viral-associated glomerulonephritis. Nephrol Dial Transplant 24:1113–1121. 19004847 10.1093/ndt/gfn627

[10] Merkle M, Ribeiro A, Wörnle M (2011) TLR3 dependent regulation of cytokines in human mesangial cells: a novel role for IP-10 and TNFa in hepatitis C associated glomerulonephritis. Am J Physiol Renal Physiol 301:57–69. 10.1152/ajprenal.00083.2011 21454254

[11] Akira S, Takeda K, Kaisho T (2001) Toll-like receptors: critical proteins linking innate and acquired immunity. Nat Immunol 2:675–680. 11477402 10.1038/90609

[12] Alexopoulou L, Holt AC, Medzhitov R, Flavell RA (2001) Recognition of double-stranded RNA and activation of NF-κB by toll-like receptor 3. Nature 413:732–738. 11607032 10.1038/35099560

[13] Matsukura S, Kokubu F, Kurokawa M, Kawaguchi M, Ieki K, et al (2007) Role of RIG-I, MDA-5, and PKR on the expression of inflammatory chemokines induced by synthetic dsRNA in airway epithelial cells. Int Arch Allergy Immunol 143 Suppl 180–83. 17541283 10.1159/000101411

[14] Vitour D, Meurs EF (2007) Regulation of interferon production by RIG-I and LGP2: a lesson in self-control. Sci STKE 384:20. 10.1126/stke.3842007pe20 17473309

[15] Yount JS, Moran TM, López CB (2007) Cytokine-independent upregulation of MDA5 in viral infection. J Virol 81 7316–7319. 10.1128/JVI.00545-07 PMC1933291 17475649

[16] Ades EW, Candal FJ, Swerlick RA, George VG, Summers S, et al (1992) HMEC-1: establishment of an immortalized human microvascular endothelial cell line. J Invest Dermatol 99:683–690. 1361507 10.1111/1523-1747.ep12613748

[17] Mannell H, Hammitzsch A, Mettler R, Pohl U, Krotz F (2010) Suppression of DNA-PKcs enhances FGF-2 dependent human endothelial cell proliferation via negative regulation of Akt. Cell Signal 22:88–96. 19781633 10.1016/j.cellsig.2009.09.015

[18] Baez S (1973) An open cremaster muscle preparation for the study of blood vessels by in vivo microscopy. Microvasc Res 5:384–394. 4709735 10.1016/0026-2862(73)90054-x

[19] Mannell H, Pircher J, Chaudhry DI, Alig SK, Koch EG, et al (2012) ARNO regulates VEGF-dependent tissue responses by stabilizing endothelial VEGFR-2 surface expression. Cardiovasc Res 93:111–119. 22002459 10.1093/cvr/cvr265

[20] Wörnle M, Schmid H, Merkle M, Banas B (2004) Effects of chemokines on proliferation and apoptosis of human mesangial cells. BMC Nephrol 5:8. 15265234 10.1186/1471-2369-5-8 PMC493268

[21] Popvic ZV, Wang S, Papatriantafyllou M, Kaya Z, Porubsky S, et al. (2011) The proteiglycan biglycan enhances antigen-specific T cell activation potentially via MyD88 and TRIF pathways and triggers autoimmune perimyocarditis. J Immunol 15; 6217–6226. 10.4049/jimmunol.1003478 PMC3428142 22095710

[22] Pircher J, Merkle M, Wörnle M, Mannell H, Ribeiro A, et al (2012) Prothrombotic effects of tumor necrosis factor alpha: implications for autoimmune disorders and their therapy with biological agents. Arthritis Res Ther 14:R225. 23079185 10.1186/ar4064 PMC3580536

[23] Terrier B, Semoun O, Saadoun D, Sène D, Resche-Rigon M, et al (2011) Prognostic factors in hepatitis C virus patients with systemic vascultitis. Arthritis Rheum 63:1748–57. 21400476 10.1002/art.30319

[24] Terrier B, Sène D, Dechartres A, Saadoun D, Ortonne N, et al (2011) Systemic vasculitis in patients with Hepatitis C virus infection with and without detectable mixed cryoglobulinemia. J Rheumatol 38:104–110. 20952479 10.3899/jrheum.100191

[25] Trejo O, Ramos-Casals M, García-Carrasco M, Cervera R, Trejo O, et al (2001) Cryoglobulinemia: study of etiologic factors and clinical and immunologic features in 443 patients from a single center. Medicine (Baltimore) 80:252–262. 11470986 10.1097/00005792-200107000-00004

[26] Antonelli A, Ferri C, Fallahi P, Ferrari SM, Sebastiani M, et al (2008) High values of CXCL10 serum levels in mixed cryoglobulinemia associated with hepatitis C infection. Am J Gastroenterol 103:2488–2494. 18775023 10.1111/j.1572-0241.2008.02040.x

[27] Antonelli A, Ferri C, Ferrari SM, Colaci M, Sansonno D, et al (2009) Endocrine manifestations of hepatitis C virus infection. Nat Clin Pract Endocrinol Metab 5:26–34. 19079271 10.1038/ncpendmet1027

[28] Feldmann G, Nischalke HD, Nattermann J, Banas B, Berg T, et al (2006) Induction of interleukin-6 by hepatitis C virus core protein in hepatitis C-associated mixed cryoglobulinemia and B-cell non-Hodgkin's lymphoma. Clin Cancer Res 12:4491–4498. 16899594 10.1158/1078-0432.CCR-06-0154

[29] Antonelli A, Ferri C, Ferrari SM, Ghiri E, Marchi S, et al (2009) High interleukin-6 and tumor necrosis factor-alpha serum levels in hepatitis C infection associated or not with mixed cryoglobulinemia. Clin Rheumatol 28:1179–1185. 19543846 10.1007/s10067-009-1218-8

[30] Kaplanski G, Maisonobe T, Marin V, Grès S, Robitail S, et al (2005) Vascular cell adhesion molecule-1 (VCAM-1) plays a central role in the pathogenesis of severe forms of vasculitis due to hepatitis C-associated mixed cryoglobulinemia. J Hepatol 42:334–340. 15710215 10.1016/j.jhep.2004.11.034

[31] Karikó K, Bhuyan P, Capodici J, Weissmann D (2004) Small interfering RNAs mediate sequence-independent gene suppression and induce immune activation by signaling through toll-like receptor 3. J Immunol 172:6545–6549. 15153468 10.4049/jimmunol.172.11.6545

[32] Kleinman ME, Yamada K, Takeda A, Chandrasekaran V, Nozaki M, et al (2008) Sequence- and target-independent angiogenesis suppression by siRNA via TLR3. Nature 452:591–597. 18368052 10.1038/nature06765 PMC2642938

[33] Farina G, York M, Collins C, Lafyatis R (2011) dsRNA activation of endothelin-1 and markers of vascular activation in endothelial cells and fibroblasts. Ann Rheum Dis 70:544–550. 21068089 10.1136/ard.2010.132464 PMC3086552

[34] Hägele H, Allam R, Pawar RD, Anders HJ (2009) Double-stranded RNA activates type I interferon secretion in glomerular endothelial cells via retinoic acid-inducible gene (RIG)-1. Nephrol Dial Transplant 24:3312–3318. 19608629 10.1093/ndt/gfp339

[35] Zimmer S, Steinmetz M, Asdonk T, Motz I, Coch C, et al (2011) Activation of endothelial Toll-like receptor 3 impairs endothelial function. Cir Res 108:1358–1366. 10.1161/CIRCRESAHA.111.243246 21493895

[36] Lundberg AM, Drexler SK, Monaco C, Williams LM, Sacre SM, et al (2007) Key differences in TLR3/poly I:C signaling and cytokine induction by human primary cells: a phenomenon absent from murine cell systems. Blood 110:3245–3252. 17660379 10.1182/blood-2007-02-072934

[37] Cocquerel L, Voisset C, Dubuisson J (2006) Hepatitis C virus entry: potential receptors and their biological functions. J Gen Virol 87:1075–1084. 16603507 10.1099/vir.0.81646-0

[38] Burlone ME, Budkowska A (2009) Hepatitis C virus cell entry: role of lipoproteins and cellular receptors. J Gen Virol 90:1055–1070. 19264629 10.1099/vir.0.008300-0

[39] Fletcher NF, Wilson GK, Murray J, Hu K, Lewis A, et al (2012) Hepatitis C virus infects the endothelial cells of the blood-brain barrier. Gastroenterology 142:634–643. 22138189 10.1053/j.gastro.2011.11.028 PMC3801216

[40] Wajant H, Pfizenmaier K, Scheurich P (2003) Tumor necrosis factor signaling. Cell Death Differ 10:45–65. 12655295 10.1038/sj.cdd.4401189

[41] Saadoun D, Resche-Rigon M, Thibault V, Piette JC, Cacoub P (2006) Antiviral therapy for hepatitis C virus-associated mixed cryoglobulinemia vasculitis: a long-term followup study. Arthritis Rheum 54:3696–3706. 17075881 10.1002/art.22168

[42] Pietrogrande M, De Vita S, Zignego AL, Pioltelli P, Sansonno D, et al (2011) Recommendations for the management of mixed cryoglobulinemia syndrome in hepatitis C virus-infected patients. Autoimmun Rev 10:444–454. 21303705 10.1016/j.autrev.2011.01.008

